# Comprehensive Comparison of Novel Bovine Leukemia Virus (BLV) Integration Sites between B-Cell Lymphoma Lines BLSC-KU1 and BLSC-KU17 Using the Viral DNA Capture High-Throughput Sequencing Method

**DOI:** 10.3390/v14050995

**Published:** 2022-05-07

**Authors:** Meripet Polat Yamanaka, Susumu Saito, Kazuyoshi Hosomichi, Yoko Aida

**Affiliations:** 1 Laboratory of Global Infectious Diseases Control Science, Department of Global Agricultural Science, Graduate School of Agriculture and Life Science, The University of Tokyo, 1-1-1 Yayoi, Bunkyo-ku, Tokyo 113-8657, Japan; meripet513@gmail.com; 2 Viral Infectious Diseases Unit, RIKEN, 2-1 Hirosawa, Wako 351-0198, Japan; kabe0912@t-net.ne.jp; 3 Department of Bioinformatics and Genomics, Graduate School of Medical Sciences, Kanazawa University, Takara-machi 13-1, Kanazawa 920-8640, Japan; khosomic@med.kanazawa-u.ac.jp

**Keywords:** bovine leukemia virus (BLV), B-cell lymphoma line, integration site, proviral DNA capture sequencing, next-generation sequencing (NGS), PCR

## Abstract

Bovine leukemia virus (BLV) infects cattle and integrates into host DNA, causing enzootic bovine leukosis (EBL), an aggressive B-cell lymphoma. Here, we developed a novel proviral DNA-capture sequencing (proviral DNA-capture-seq) method investigating BLV proviral integration in two B-cell lymphoma lines, BLSC-KU1 and BLSC-KU17, derived from BLV-infected cattle with EBL. We designed BLV-specific biotinylated probes to capture the provirus genome and enrich libraries for next-generation sequencing. Validation showed high specificity and efficient enrichment of target sequence reads as well as identification of three BLV proviral integration sites on BLV persistently infected FLK-BLV cells as a positive control. We successfully detected a single BLV proviral integration site on chromosome 19 of BLSC-KU1 and chromosome 9 of BLSC-KU17, which were confirmed by standard PCR and Sanger sequencing. Further, a defective provirus in BLSC-KU1 and complete BLV proviral sequence in BLSC-KU17 were confirmed using long PCR and sequencing. This is the first study to provide comprehensive information on BLV proviral structure and viral integration in BLSC-KU1 and BLSC-KU17. Moreover, the proposed method can facilitate understanding of the detailed mechanisms underlying BLV-induced leukemogenesis and may be used as an innovative tool to screen BLV-infected cattle at risk at an earlier stage than those that have already developed lymphoma.

## 1. Introduction

Bovine leukemia virus (BLV) is an exogenous retrovirus that causes enzootic bovine leukosis (EBL), a lymphoma of infected CD5^+^ IgM^+^ B cells, in cattle. BLV naturally infects cattle and water buffaloes, and experimental infection has been demonstrated in other species and cell lines [1]. However, BLV is known to induce lymphoma only in cattle and sheep [1]. BLV induces a life-long persistent infection that generally remains asymptomatic [1]. In infected cattle with no evident tumors, BLV has been identified in B cells, CD2^+^ T cells, CD3^+^ T cells, CD4^+^ T cells, CD8^+^ T cells, γ/δ T cells, monocytes, and granulocytes [2,3,4,5]. However, a small proportion (up to 5%) of BLV-infected cattle develop lymphoma originating from mono- or oligoclonal accumulation of CD5^+^ IgM^+^ B cells after a relatively long period of latency. BLV is primarily transmitted through the transfer of infected lymphocytes via both horizontal and vertical routes [1]. Iatrogenic factors also play a significant role [1,6]. The clinical signs of BLV-induced tumors vary and primarily include digestive disturbances, weight loss, weakness, reduced milk production, loss of appetite, and enlarged lymph nodes [7]. BLV entry into cells is initiated by interaction between the envelope glycoprotein and host cellular receptor, cationic amino acid transporter 1/solute carrier family 7 member 1, wherein viral RNA is released into the cytoplasm and catalyzed by viral-encoded protein reverse transcriptase to produce viral DNA, which is then translocated into the cell nucleus where it is integrated into the cellular genome as a provirus through the catalytic function of another viral-encoded enzyme, integrase [1,8]. The BLV provirus remains in the cellular genome throughout the disease course, in the absence of detectable BLV antibodies [9]. The integrated provirus is transcribed and serves as a template for producing new viral particles. Thus, integration of the BLV provirus into the host genome is an essential step in the BLV life cycle that enables viral persistence and induces B-cell lymphoma in cattle. Like other retroviruses, including HTLV-1, BLV spreads within the host by either an infectious cycle that takes place in the initial phase of infection, or a mitotic cycle in which BLV maintains or increases its proviral copy number by the clonal expansion of infected cells, especially in the chronic phase of infection [10,11]. Therefore, the proviral sequences are extremely stable.

The bovine B-cell lymphoma lines BLSC-KU1 and BLSC-KU17 were established from leukemic cells of BLV-infected cattle with EBL. Both cell lines grow as single, free-floating cells that do not attach to a glass surface [12,13,14]. These cell lines have B-cell surface marker and tumor-associated antigen on their cell surface [12,13,14,15,16]. BLSC-KU1 is a morphologically lymphoblastic cell line that contains a single defective BLV provirus, with a partial region spanning the *pol* and *env* genes in the genome deleted and no production of BLV or BLV antigen [13]. Further, BLSC-KU1 cells form solid tumors in nude mice [13,17]. In contrast, the BLSC-KU17 cells can produce BLV, indicating that this cell line may contain a complete BLV provirus in the cellular genome; however, it does not form of tumors in nude mice [14]. However, the whole sequence and genomic structure of the BLV provirus integrated in both BLSC-KU1 and BLSC-KU17 cells have not been determined.

Most effort has been devoted to the analysis of proviral integration sites in other retroviruses, such as human immunodeficiency virus type-1 (HIV-1) [18,19,20,21] and human T-cell leukemia virus type-1 (HTLV-1) [10,11,22], and the results indicate that these viruses are preferentially integrated into genes with active transcription [10,23,24]; further, both HIV-1- and HTLV-1-infected cells can undergo clonal expansion, which is enhanced by the integration of its provirus in actively transcribed areas of the genome [10,21,25,26,27,28]. However, the distribution of BLV proviral integration sites and quantification of clonal expansion in infected cells remains unexplored [29]. Thus, the impact of BLV integration sites and clonal expansion on lymphoma development remains to be studied in detail.

Previous studies have shown the applicability of retroviral sequences enrichment using proviral-specific biotinylated probes in the integration-site analysis of retroviruses [29,30,31,32]. Randomly fragmented DNA containing the junction of provirus–host DNA can be obtained and enriched using virus-specific probes through viral DNA-capture enhancement, and then subjected to analysis of the proviral integration site through high-throughput sequencing. Here, we developed a new BLV proviral DNA-capture-sequencing (proviral DNA-capture-seq) method and applied it to the bovine B-cell lymphoma lines BLSC-KU1 and BLSC-KU17 to provide comprehensive information regarding BLV integration sites in these cell lines. We successfully identified a single BLV proviral integration site in both cell lines and confirmed the integration sites using standard PCR.

## 2. Materials and Methods

### 2.1. Cell Lines and Extraction of Genomic DNA

The bovine B-cell lymphoma lines BLSC-KU1 and BLSC-KU17 were established from lymphoma cells of BLV-infected cattle with EBL. KU1 cells were maintained in Dulbecco’s modified Eagle’s medium (DMEM) (Thermo Fisher Scientific, Waltham, MA, USA) supplemented with 20% fetal bovine serum (FBS) (Sigma-Aldrich, St. Louis, MO, USA) [13], whereas KU17 was maintained in DMEM supplemented with 10% FBS, both at 37 °C [14]. Positive control (FLK-BLV) cells with a permanent BLV infection [33] were established from fetal lamb kidney monolayers after in vitro infection with cell-free BLV preparation and serial passage [33]. FLK-BLV cells were cultured in DMEM (Thermo Fisher Scientific) containing 10% heat-inactivated FBS (Sigma-Aldrich).

Genomic DNA was extracted from all cell lines using the Wizard Genomic DNA purification kit (Promega Corporation, Tokyo, Japan), according to the manufacturer’s instructions. The extracted DNA was stored at −20 °C until required.

### 2.2. PCR Amplification of BLV Proviral Genome and Sequencing

The integrated BLV proviral genome of BLSC-KU1 and BLSC-KU17 were amplified by PCR using PrimeSTAR GXL DNA Polymerase (Takara Bio Inc., Kusatsu, Japan) and specific primers (Life Technologies Japan Ltd., Tokyo, Japan) targeting the BLV genomes [34,35], as shown in Table S1. The PCR conditions were similar to those reported in previous publications, with small modifications [34,35]. The final reaction mixture (25 μL) contained 5 μL of 5 × PrimerSTAR GXL Buffer, 2 μL of 2.5 mM dNTP mix, 1 μL of each primer (each at 10 pmol), 2 μL of template (50 ng/μL), and 0.5 μL PrimerSTAR GXL DNA Polymerase. PCR amplification was performed as follows: 98 °C for 2 min, followed by 33 cycles of denaturation at 98 °C for 10 s, annealing at 60 °C for 30 s, and extension at 68 °C for 1 min/kilo-base. The PCR products were electrophoresed on agarose gel (0.8–3%) and purified using a FastGene Gel/PCR Extraction Kit (Nippon Genetics Co., Ltd. Tokyo, Japan). The purified PCR products were sequenced on an ABI3730xl DNA Analyzer using an ABI PRISM Big Dye Terminator v 3.1 Ready Reaction Cycle Sequencing Kit (Applied Biosystems, Foster City, CA, USA). The BLV genome was obtained by sequencing overlapping genomic fragments.

### 2.3. BLV Proviral DNA-Capture-Seq Method

**Figure 1:**
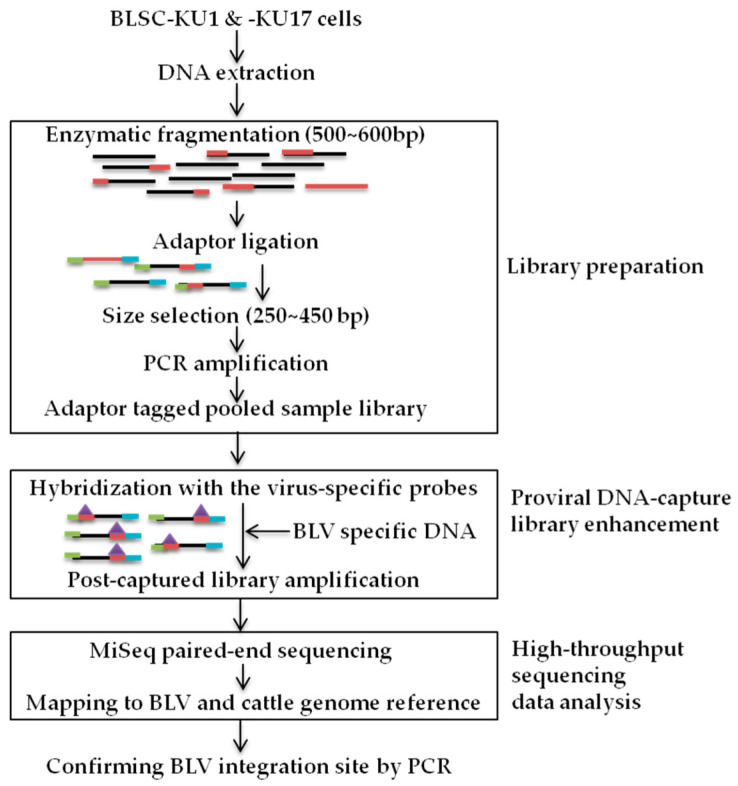
Schematic of BLV proviral DNA-capture-sequencing (proviral DNA-capture-seq) to detect the integration sites of BLV provirus in BLSC-KU1 and BLSC-KU17 cell lines, such as library preparation, proviral DNA capture library enhancement, and high-throughput sequencing data analysis.

The method of proviral DNA-capture-seq is shown in Figure 1.

#### 2.3.1. DNA Probe

For library enrichment, five biotinylated probes (Thermo Fisher Scientific, Yakohama, Japan) were custom-designed on the basis of the BLV reference sequence, FLK-BLV (accession number EF600696) (Table S2). These probes were 100 bp in length, targeting the LTR, *gag*, and *tax* regions. The concentration of all probes was 100 μM, and they were stored at −20 °C until used, as recommended by the manufacturer. Virus–host chimeric DNA fragments and BLV proviral genome-only fragments were captured using these specific biotinylated DNA probes.

#### 2.3.2. Library Preparation

Genomic DNA (1 μg) was enzymatically fragmented to an average length of approximately 500–600 bp using the KAPA Hyper Plus kit (Roche, Cape Town, South Africa), following end repair and addition of adenosine at the 3′-ends of fragmented DNA. For sample traceability and library amplification, 5 μL each of 24 barcoded SeqCap index adaptors (kit A and kit B, 12 index adaptors per kit) (Roche Sequencing Solutions, Inc., Pleasanton, CA, USA) was ligated to DNA fragments of each individual sample using the KAPA Hyper Plus kit according to the manufacturer’s instructions. Adaptor-tagged library fragments were purified using Agencourt AMPure XP beads (Beckman Coulter, Inc., Brea, CA, USA) on the NGS Magna Stand Ch YS-Model (Nippon Genetics Co., Ltd.) according to the manufacturer’s instructions. In order to remove unwanted fragment sizes and low molecular weight material, such as adaptor dimers, that interfere with downstream library preparation steps (including cluster formation, sequencing, and analysis), double-sided size selection to keep fragments from 250 bp to 450 bp was performed using Agencourt AMPure XP beads before the adaptor-tagged purified libraries were subjected to PCR amplification.

#### 2.3.3. Targeted Enrichment

After library synthesis, two test samples plus the control (and three samples from another experiment), containing different index sequences, were mixed together to reach the recommended amount of 1 μg, and used for the enrichment step involving hybridization with the virus-specific probes. This was performed using the SeqCap EZ Hybridization and Wash Kit (Roche NimbleGen, Pleasanton, CA, USA) following the instructions of the protocol for DNA probe hybridization and target capture. Briefly, library DNA was first mixed with SeqCap EZ developer reagent and hybridization-enhancing oligo (Roche Sequencing Solutions, Inc., San Jose, CA, USA), followed by purification using Agencourt AMPure XP beads (Beckman Coulter, Inc.). The post-purified library DNA mixture was then dissolved in hybridization buffer. After an incubation step of 10 min at 95 °C, the probes were added for hybridization at 47 °C for 12 h. SeqCap Pure Capture beads (Roche Sequencing Solutions, Inc.) were added to the hybridization mixture, and the sample was additionally incubated for 45 min at 47 °C. After the recommended washing steps, the captured DNA was amplified by PCR and further purified using Agencourt AMPure XP beads (Beckman Coulter, Inc.). DNA libraries enriched for proviral sequences were quantified by qPCR using the Illumina P5 and P7 primers prior to sequencing. The quality and quantity of the amplified libraries were assessed using an Agilent 2100 Bioanalyzer (Agilent Technologies, Waldbronn, Germany) and DNA 1000 Assay.

#### 2.3.4. High-Throughput Sequencing Data Analysis

Multiplexed enriched libraries were subjected to cluster generation using the MiSeq Reagent Kit v3 (600 cycles) (Illumina, Inc., San Diego, CA, USA). Raw sequences were generated as Fastq using Illumina MiSeq with 600 cycles of paired-end reads, and were validated by evaluating the distribution of quality scores. Validated fastq files were aligned using the Burrows–Wheeler Aligner tool (BWA v. 0.7.8-r455) [36] against a reference sequence of BLV (accession number: EF600696) with or without the cattle reference genome (Bos_taurus_UMD_3.1/bos Tau8) (Bos_taurus_UMD_3.1.1-Genome-Assembly-NCBI (nih.gov, accessed on 5 May 2022)) using the BWA-MEM algorithm31. The resulting alignment map (SAM) format output was suitable for analyses using SAMTools [37]. Sequence quality, depth of coverage, and short-read alignment were analyzed using SAMTools [37]. PCR duplicates were removed using the Picard (http://broadinstitute.github.io/picard/; accessed on 5 May 2022). The mapped data were visualized using Integrative Genomics Viewer software (IGV) [38]. To calculate the error rate of the sequencing step, we individually analyzed the sequencing quality score (Q-score) of each base read.

### 2.4. Integration Site Analysis

When retroviruses are integrated into the host genome, short repetitive host sequences are generated adjacent to the proviral genome, such as a 6 bp duplication of the host sequence in HTLV-1 integrated sites [39]. In addition, there are no proviral sequences in the reference cow genome, only the host sequences of virus–host reads were aligned with either upstream or downstream sequences of the viral integration sites. To determine the candidate integration sites of the proviruses, virus–host chimeric reads fulfilling the following conditions were selected: (1) presence of the distinctive 6–7 bp duplication of the host sequence generated during the integration, and these repeated host genome sequence were overlapped to each other when pair-end reads were aligned and mapped to host reference genome; (2) alignment of the bovine portion of at least one “left reads” and “right reads” to the same chromosome with a convergent orientation.

### 2.5. Confirmation of Provirus Integration Sites by Sanger Sequencing

To confirm the BLV integration sites, the BLV–host genome junction was amplified using PrimeSTAR GXL DNA Polymerase (Takara Bio Inc.). PCR was performed using two primes, one targeting the host genome and the other targeting the BLV region, as shown in Table S3. The final reaction mixture (20 μL) contained 4 μL of 5× PrimerSTAR GXL Buffer, 1.6 μL of 2.5 mM dNTP mix, 0.6 μL of each primer (each at 10 pmol), 2 μL of template (50 ng/μL), and 0.4 μL of PrimerSTAR GXL DNA Polymerase. PCR amplification was performed as follows: 98 °C for 2 min, followed by 40 cycles of denaturation at 98 °C for 15 s, annealing at 60 °C for 15 s, and extension at 68 °C for 30 s/kbp. The PCR products were purified using Exo-SAP IT (USB Corp., Cleveland, OH, USA) and sequenced on an ABI3730xl DNA Analyzer with the same PCR primers, using an ABI PRISM Big Dye Terminator v. 3.1 Ready Reaction Cycle Sequencing Kit (Life Technologies). The obtained sequences were searched using BLAST against BLV references sequences and the cattle genome (Bos_taurus_UMD_3.1/bos Tau8) using NCBI BLAST and Genome Browser.

## 3. Results

### 3.1. PCR Amplification and Whole Genome Sequencing of BLV Provirus in BLSC-KU1 and BLSC-KU17 Cell Lines

**Figure 2:**
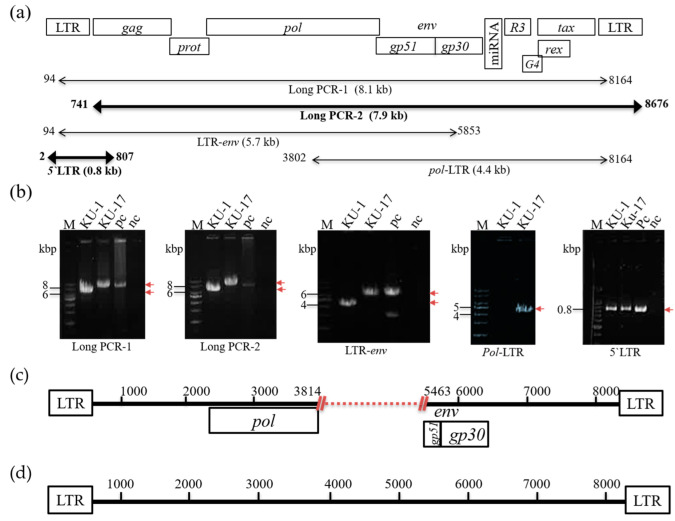
Schematic presentation of PCR amplification and sequencing of BLV provirus in BLSC-KU1 and BLSC-KU17 cell lines. (**a**) Proviral structure of BLV in the FLK-BLV subclone pBLV913, complete genome (accretion number: EF600696), primer pairs, and the lengths of expected PCR products. Numbers indicate the start and end sites of primer binding sites; numbers under the bar with kbp indicate the length of expected PCR products; and dark bars indicate the PCR products subjected to sequencing. (**b**) Electrophoresis images of amplified PCR products. M, molecular weight marker (1 kbp and 100 bp DNA Ladder), numbers with lines indicate the size of the DNA ladder, and arrows in red indicate the target PCR product; nc, no-template DNA-negative control (water substituted for DNA template); pc. DNA from FLK-BLV cell line. (**c**,**d**) Visualization of proviral sequences results for BLSC-KU1 (**c**) and BLSC-KU17 (**d**). Numbers denote the positions of complete sequences of the FLK-BLV subclone pBLV913. Dot with red indicates the deleted portion.

Although the bovine B cell lymphoma lines BLSC-KU1 and BLSC-KU17 were established from leukemic cells of BLV-infected cattle with EBL [12,13,14], the complete sequences and genomic structure of the BLV provirus integrated in both cell lines remain unknown. Therefore, to characterize the whole genome sequence of the BLV provirus integrated in BLSC-KU1 and BLSC-KU17 cell lines, we first performed two sets of long PCR and three sets of short PCR (Figure 2a). The electrophoresis result in BLSC-KU17 showed a single band with the expected length that was the same as that of the positive control FLK-BLV in each of the five sets of PCR amplifying the long and short regions of BLV, such as long PCR-1 (target 8.1 kbp), long PCR-2 (target 7.9 kbp), LTR-*env* (amplifying from 5′-LTR to partial *env*: target 5.7 kbp), *pol*-LTR (amplifying from partial *pol* to 3′-LTR; target 4.4 kbp), and 5′-LTR (target 0.8 kbp) (Figure 2b). Thus, our results implied that the complete BLV provirus genome was integrated into BLSC-KU17 cells. In contrast, in BLSC-KU1 cells, band sizes smaller than the expected lengths were observed in three different single DNA fragments of approximately 6.6, 6.3, and 4 kbp after amplification by long PCR-1, long-2, and LTR-*env*, respectively. Additionally, no DNA bands were detected in the *pol*-LTR PCR set (Figure 2b). These results clearly demonstrated that the BLV provirus in BLSC-KU1 is not a full genome and that the BLV provirus integrated in BLSC-KU1 has a 1.7 kbp deletion.

To further confirm the genomic structure of the BLV provirus integrated in BLSC-KU1 and BLSC-KU17, we performed whole genome sequencing using the PCR product of long PCR-2 and 5′-LTR. As expected, the sequencing results clearly confirmed that the deletion of BLV provirus (7063 bp) in BLSC-KU1 was from nucleotide position (nt) 3814 to nt 5463, spanning the C-terminal of *pol* and most of the *env-gp51* gene (accession number: LC682198) (Figure 2c). The failure of *pol*-LTR PCR amplification can be explained by the sequence result showing a deletion in part of the *pol* gene around the PCR primer binding position. Our sequencing results are in agreement with Southern blot analysis of a previous publication [13]. In contrast, we obtained the full BLV genome sequence (8714bp) of BLSC-KU17, as schematically presented in Figure 2d (accession number: LC681771).

### 3.2. Development of the Proviral DNA-Capture-Seq Method

**Figure 3:**
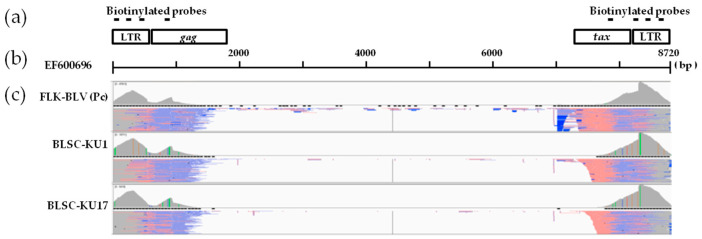
Visualization of paired-end short read sequences mapped to the BLV reference FLK-BLV sequence (EF600696). (**a**) The schematic structure of the biotinylated probe targeting region. Horizontal lines indicate biotinylated probes. (**b**) The schematic length of the BLV reference FLK-BLV sequence (EF600696). (**c**) The visualization of the mapping of paired-end reads of positive control FLK-BLV, and BLSC-KU1 and BLSC-KU17 cell lines obtained using next-generation sequencing.

Next, we developed a novel proviral DNA-capture-seq method to investigate BLV proviral integration in BLSC-KU1 and BLSC-KU17 cells, as shown in Figure 1. To efficiently enrich BLV sequences and host genome sequences of interest, we first custom-designed DNA probes targeting the BLV-LTR, *gag*, and *tax* regions, on the basis of the complete genome of the FLK-BLV subclone pBLV913 (EF600696) (Table S2). The genomic DNA was enzymatically fragmented to an average length of approximately 600 bp to generate DNA libraries. The resulting adaptor-ligated DNA libraries were hybridized to five custom-designed biotinylated probes, three probes targeting LTR, one targeting *gag*, and another targeting the *tax* gene; these were subsequently captured by streptavidin beads. The proviral DNA-capture-seq method was then applied to positive control (DNA from ovine FLK-BLV cells, which are permanently infected with BLV). FLK-BLV generated 5.82 million raw reads, of which 4.55 million raw reads were mapped to the target BLV reference genome (EF600696), with a ratio of 78.2% (Table 1). Sequence reads mapped the target region of the BLV reference genome were visualized, as shown in Figure 3. Then, BLV-host chimeric reads were mapped to ovine host genome (the Ovine Genome Assembly Oar_v4.0). Five BLV-specific probes were used in this study, and although the enrichment of the probe covering *tax* was low, the proportion of paired-end reads mapped to BLV was relatively high in the remaining four probes. The custom-designed biotinylated probes used in this study were shown to have high capture efficiency and specificity for targeting the BLV genome and virus–host chimeric DNA fragments.

**Figure 4:**
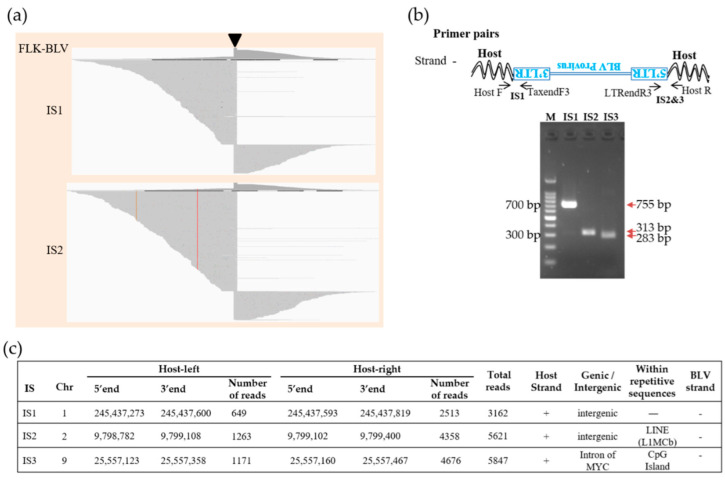
Visualization of BLV integration sites (ISs) in FLK−BLV cells. (**a**) Visualization of NGS reads mapped to the ovine genome around the ISs. The arrow−head indicates the location of the IS. Sequences of virus−host chimeric reads next to the end of BLV gnome are indicated as host−left and host−right; respectively. (**b**) Confirmation of detected ISs by conventional PCR. Host indicate ovine FLK cellular genome, the upside−down writing of BLV provirus and LTR indicates that negative strand BLV provirus was integrated. M, molecular weight marker (100−bp DNA Ladder). Numbers besides DNA ladder indicate ladder size. Numbers with arrows in red indicate the target PCR product length. (**c**) Detailed information of ISs in FLK−BLV cells.

We analyzed the BLV integration sites in FLK-BLV cells. FLK-BLV cells have often been used as controls in studies related to BLV; previous studies have showed multiple copies of the BLV genome integrated into the genome of the FLK-BLV cells by either Southern blotting analysis [2,33] or target enrichment next-generation sequencing (NGS) [29]. The five biotinylated probes used in this study efficiently and specifically captured the BLV provirus and proviral–host chimeric DNA fragments in the DNA library of FLK-BLV cells. We detected three BLV integration sites in the FLK-BLV cells by analyzing host sequences in the provirus–host chimeric reads. Visualization of the sequences of paired-end reads mapped to the host genome showed a peak between NGS reads in two of the detected integration sites (Figure 4a). BLV integration sites (ISs) detected in the FLK-BLV cells were confirmed by PCR amplification of provirus–host chimeric sequences using primer sets targeting both BLV and the host genome linked with BLV (Figure 4b). All of the three detected BLV integration sites showed high read depths (Figure 4c). Thus, the newly developed viral DNA capture target enrichment high-throughput sequencing was successfully performed.

### 3.3. Identification of Integration Site by Proviral DNA-Capture-Seq in BLSC-KU1 Cells and Its Confirmation

**Figure 5:**
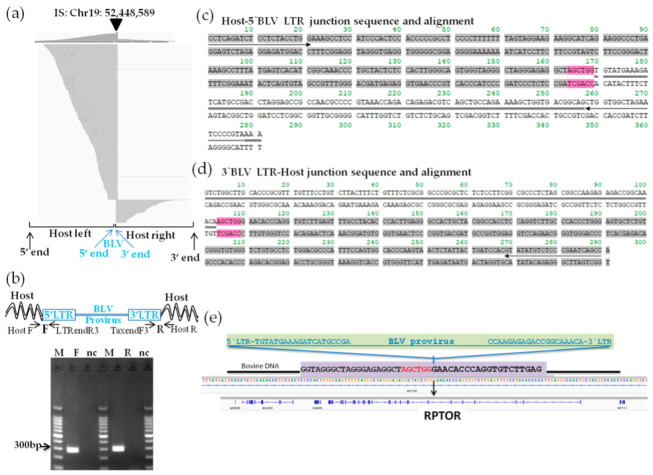
Visualization of next-generation sequencing (NGS) reads mapped to the bovine genome around the integration site of BLSC-KU1 and confirmation of its provirus integration sites. (**a**) Paired-end read mapping and visualization of host genome sequences in which the BLV provirus is integrated in BLSC-KU1. The arrow-head indicates the location of the integration site. The sequences of virus–host chimeric reads next to the end of the BLV genome are indicated as host-left and host-right, respectively. (**b**) Confirmation of detected integration sites by electrophoresis of PCR amplicons. Arrow indicates forward (F) and reverse (R) primer binding sites corresponding to cattle and BLV proviral sequences. M, molecular weight marker (100-bp DNA Ladder); nc, no-template DNA-negative control (water substituted for DNA template). (**c**,**d**) Sequence result of host–5′-BLV LTR (**c**) and 3′-BLV LTR–host (**d**) junction DNA in BLSC-KU1 cells. Sequences with gray color indicate host cattle genome; sequences with double line indicate the BLV provirus sequence; 6 bp nucleotide sequences colored in pink indicate duplicated host cattle sequences. Arrow indicates primer binding site. (**e**) Schematic presentation of BLV integration in the intron of regulatory-associated protein of mTOR (RPTOR) of chromosomal (Chr)19 nt 52,448,589 in KU1 cell lines.

To clarify the BLV proviral integration sites in both tested cell lines, we first applied the newly developed proviral DNA-capture-seq method to BLSC-KU1 cells. As shown in Table 1, BLSC-KU1 generated 5.06 million raw reads, of which 4.08 (80.6%) million raw reads were mapped to the target BLV reference genome (EF600696). Paired-end reads were first aligned against the BLV reference FLK-BLV (EF600696) (Figure 3), and then to the cow host reference genome (Bos_taurus_UMD_3.1/bos Tau8) and were visualized using IGV (Figure 5).

After mapping the virus–host paired-end reads to the cattle reference, we successfully detected a large number of reads with high depth (8100 reads), mapped to chromosome (Chr) 19 and centering nucleotide position 52,448,589 in the bovine genome of BLSC-KU1 cells (Figure 5a, Table 2). Through BLAST analysis of paired-end read sequences using the Gene browser BLAT tool and NCBI nucleotide BLAST, BLV was found to be integrated in sense orientation with the host genome of BLSK-KU1 cells. We did not observe any other possible candidate sites in BLSK-KU1, except for BLV integration site in Chr19, indicating that BLSK-KU1 originates from the monoclonal expansion of BLV-infected B cells. This result was also consistent with a previously reported Southern blotting analysis [13].

Next, we confirmed the NGS data of the BLV integration site in BLSK-KU1: the candidate integration site was subjected to PCR amplification using a pair of primers, one targeting the host genome and another targeting the integrated BLV proviral genome of interest. Therefore, we designed primer pairs, named Chr19F and BLV-LTRendR3 for the host genome-5′-BLV LTR junction, and Chr19R and BLV-TaxendF3 for the BLV 3′-LTR-host genome junction. The primer sequences are shown in Table S3. These primers were paired because BLV was integrated into BLSC-KU1 in sense orientation with the host genome. As shown in the electrophoresis result in Figure 5b, we detected a target band of 300 bp in the host–BLV junctions at both the forward and reverse primers. Specific target bands were subjected to Sanger sequencing using the same PCR primers. Host–BLV junction sequences were successfully obtained at both ends of the LTR, as shown in Figure 5c,d, and were separately aligned to the cow reference genome (Bos_taurus_UMD_3.1/bos Tau8) using Genome Browser BLAST tools and the BLV provirus genome sequences using NCBI nucleotide BLAST. We also observed 6 bp of short repetitive host genome sequences around the BLV provirus integration (colored pink in Figure 5c,d). Furthermore, BLV integration occurred within the intron of the regulatory-associated protein of mTOR (*RPTOR*) on Chr19 nt 52,448,589 in BLSC-KU1, as shown in Figure 5e and Table 2.

### 3.4. Identification of the Integration Site in the BLSC-KU17 Cell Line and Its Confirmation by Proviral DNA-Capture-Seq

**Figure 6:**
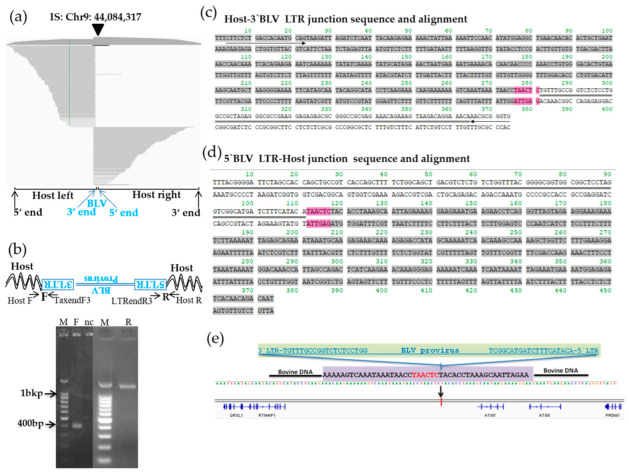
Visualization of next-generation sequencing (NGS) reads mapped to the bovine genome around integration site of the BLSC-KU17 and confirmation of its provirus integration sites. (**a**) Paired-end reads mapping and visualization of host genome sequences in which the BLV provirus is integrated in BLSC-KU17 cells. The arrow-head indicates the location of the integration site. The sequences of virus–host chimeric reads next to the end of the BLV genome are indicated as host-left and host-right, respectively. (**b**) Confirmation of the detected integration site by electrophoresis of the PCR amplicon. Arrow indicates forward (F) and reverse (R) primer binding sites corresponding to cattle and BLV proviral sequences. M, molecular weight marker (100-bp DNA Ladder); nc, no-template DNA-negative control (water substituted for DNA template). (**c**,**d**) Sequence result of host–3′-BLV LTR (**c**) and 5′-BLV LTR–host (**d**) junction DNA in BLSC-KU17 cells. Arrow indicates the primer binding site. Sequences in gray color indicate host cattle genome; sequences with double line indicate the BLV provirus sequence; 6 bp nucleotide sequences colored in pink indicate duplicated host cattle sequences. (**e**) Schematic presentation of the BLV integration site in BLSC-KU17 cells.

We applied the newly developed proviral DNA-capture-seq method to BLSC-KU17 cells. BLSC-KU17 generated 5.07 million raw reads, of which 80.9% of reads (4.1 million) were mapped to the target BLV reference genome (EF600696) (Table 1, Figure 3). As far as KU17 is concerned, we observed mapping of paired-end reads in repeatMasker L1_BT of LINE on Chr9 at nt 44,084,317 aligned to the cow reference genome (Bos_taurus_UMD_3.1/bos Tau8) with high depth (956 reads), as visualized using IGV (Figure 6a, Table 2). Further, we did not observe any other possible candidate sites, except the BLV integration site in the host genome Chr9, indicating that BLSC-KU17 contained a single complete BLV provirus. BLAST analysis of the paired-end read sequences revealed that BLV was integrated in the antisense orientation with the host genome in BLSK-KU17 cells.

To further confirm the BLV integration site detected by NGS in Chr9 of BLSC-KU17, we designed primers targeting both the host and proviral genome as Chr9F and BLV-TaxendF3 for the host genome 3′-BLV LTR junction, and Chr9R and BLV-LTRendR3 for 5′-BLV LTR–host genome junction (Table S3). These primers were constructed on the basis of antisense orientation of BLV with the host genome. After PCR amplification, we observed about 400 bp and 1.4 kbp of specific target bands in Figure 6b and subjected them to Sanger sequencing using the same PCR primers. Host–BLV junction sequences were successfully obtained as shown in Figure 6c,d. Then, sequences were separately aligned to either the cattle reference genome (Bos_taurus_UMD_3.1/bos Tau8) through Genome Browser Blat tools, or BLV provirus genome sequences through NCBI nucleotide Blast. A total of 6bp of duplicated host sequences were also observed (colored pink in Figure 6c,d). BLV was integrated into the intergenic region downstream of *RTN4IP1* and upstream of *ATG5* (Figure 6e, Table 2).

## 4. Discussion

We drew three major conclusions from the results of this study on BLV integration in two B-cell lines established from BLV-infected cattle with EBL. First, we developed a new BLV proviral DNA-capture-seq method, in which fragments with viral sequences in DNA libraries are captured using proviral DNA biotinylated probes targeting BLV-LTR, *gag*, and *tax*, and sequenced them by high-throughput sequencing. Evaluation of this method showed efficient enrichment of target sequence reads and integration of the BLV proviral genome in persistently infected FLK-BLV cells. Our visualization of sequences of paired-end reads mapped to the host genome in DNA from FLK-BLV cells showed a pattern similar to that reported in a previous publication [29]. Secondly, we found evidence for the first-time regarding two BLV integration sites in BLSC-KU1 and BLSC-KU17 cells using our newly developed proviral DNA-capture-seq. As summarized in Table 2, our results clearly show that the BLV provirus in BLSC-KU1 is integrated at a single site in the intron of the RPTOR gene, whereas that in BLSC-KU17 is integrated at a single site in the intergenic region between RTN4IP1 and ATG5. Although it has been previously shown that BLV preferentially integrates in transcriptionally active genomic regions adjacent to cancer drivers [40], the proviral integration sites varied between samples, and no clear evidence of recurrent proviral integration was observed. Thus, the integration sites detected in this study are different from those reported in previous studies wherein BLV integration sites in EBL cattle were found in retroelements, such as short interspersed nuclear element (SINE), long interspersed nuclear element (LINE), and long terminal repeat (LTR) of the endogenous retrovirus [41], as well as Refseq genes including Family with sequence similarity 92 member A (FAM92A), Ankyrin 3 (ANK3), and uncharacterized genes [29]. In contrast, in T-cell lymphomas induced by Moloney murine leukemia, provirus integration in a single locus activates the expression of multiple genes, some of which may be located at a long distance from the site of integration [42]. Therefore, further studies are needed to clarify the impact of BLV proviral integration on the expression and function of *RPTOR*, *RTN4IP1*, and *ATG5*, the host cellular genes reported in this study. Third, even though BLV structures were studied in these two cell lines by Southern blotting analysis, the current study is the first to provide new information on the defective and full proviral genome sequences in BLSC-KU1 and BLSC-KU17 cell lines, respectively, as well as to confirm the exact deletion regions of the defective BLV provirus in BLSC-KU1 cells through long PCR and sequencing (Table 2). Further, the present study showed that both cells contained a single BLV provirus in the cellular genome. Thus, integration of BLV provirus in both cell lines occurs at a single site within the host genome, as determined by proviral DNA-capture-seq.

In this study, we custom-designed five BLV specific probes, three targeting BLV-LTR, one targeting *gag*, and another targeting *tax* region, in order to efficiently enrich BLV and BLV–host chimeric genome sequences of interest for investigating the BLV integration site. The BLV genome is 8.7 kbp [1] and is extremely small compared to the sheep and cow host genome sequences. To efficiently obtain the BLV sequences and BLV–host chimeric genome sequences, we first filtered the NGS data by aligning and mapping all reads to the BLV reference genome (EF600696). Filtered ratios were 80.6% and 80.9%, respectively (Table 1), after which the filter-obtained data were again mapped to host genome. Among the filtered-lost genome mapped data, 8100 and 956 of BLV–host chimeric reads were mapped to the BLV integration site in BLSC-KU1 and BLSC-KU17, respectively (Table 2), indicating the very high efficiency of target enrichment and the specificity of probes in BLV integration site detection. In a previous study, Ohnuki et al. used 145 custom-designed xGen Lockdown Probes (IDT) to obtain 0.1 million reads that covered the entire BLV proviral sequence and contained BLV–host chimeric genome sequences [29]. Compared with their result, even though we only used five biotinylated BLV probes, we obtained 4.08 and 4.10 million reads mapping to the BLV reference genome, and the depth of BLV–host chimeric reads mapped to BLV integration sites of the host genome were relatively high (Table 2). Moreover, the five custom-designed probes are economically less expensive. However, the probe in the *tax* region has a lower enrichment ratio than other probes and thereby it needs to improve its enrichment ratio by changing the design or increasing the ratio of the amount of *tax* probe to other probes.

The mechanisms underlying BLV-induced leukemogenesis have not yet been fully elucidated. Viral products such as Tax are thought to play significant roles in oncogenic mechanisms. However, Tax may only induce immortalization of CD5^+^ IgM^+^ B-cells among BLV-infected B cells including CD4^+^ T cells, CD8^+^ T cells, γ/δ T cells, monocytes, and granulocytes in cattle, thereby conferring a selective transformation advantage to the infected CD5^+^ IgM^+^ B cells by a second event [1]. Previous studies have demonstrated that polymorphisms [43,44,45], mutations [46,47], or gene expression alternations [48,49] in the host genome are involved in the risk of developing lymphoma. Moreover, BLV integration into the host genome itself would accelerate the proliferation of infected cells [11,40]. In this study, we identified candidate host genes that may contribute to BLV-induced leukemogenesis in two B-cell lymphoma lines, BLSC-KU1 and BLSC-KU17. One of the most important findings of our study was that we detected and confirmed the BLV proviral integration site at Chr19 nucleotide position 52,448,589 in BLSC-KU1. The BLV provirus was integrated into the intron of the *RPTOR* gene in Chr19 of BLSC-KU1. According to publications, the *RPTOR* gene encodes a protein involved in the mTOR signaling pathway that responds to nutrient and insulin levels for regulating cell growth [50,51] and is involved in mRNA translation and autophagy [52]. RPTOR is altered in 1.77% of all cancers, with lung adenocarcinoma, colon adenocarcinoma, breast invasive ductal carcinoma, cutaneous melanoma, and endometrial endometrioid adenocarcinoma having the greatest prevalence of alterations [53]. Therefore, further research on the interaction between RPTOR and BLV proviral integration and its impact on BLV-induced leukemogenesis is indispensable.

Another finding of our study is that in the KU17 cell line, the BLV provirus was integrated in the intergene region between *RTN4IP1* and *ATG5*, distantly close to *ATG5*. *RTN4IP1* encodes reticulon-4-interacting protein 1 (RTN4IP1), which is involved in regulating ganglion cell neurite growth [54]. *ATG5* encodes autophagy-related 5 protein, which combines with autophagy protein 12 and functions as an E1-like-activating enzyme in a ubiquitin-like conjugating system [55]. The encoded protein is involved in several cellular processes, including lymphocyte development and proliferation [56], MHC II antigen presentation, adipocyte differentiation, apoptosis [57,58], lymphocyte development, and B- and T-cell survival and proliferation [59,60]. In addition, downregulation of ATG5 protein and mutations in the ATG5 gene have also been linked with prostate [61] and colorectal cancers [62]. Therefore, further comprehensive studies are required to define the effect of BLV integration on downstream genes such as ATG5 and its impact on BLV-induced leukemogenesis.

We showed a defective provirus in BLSC-KU1, with approximately 1.7 kbp deleted from the C-terminal of the *pol* gene to most of the *env-gp51* gene (nucleotide position: nt 3814 to nt 5463). This result was consistent with previous studies showing that BLSC-KU1 cells contain a defective provirus showing deletion of a partial region spanning the *pol* and *env* genes by Southern blotting [12]. Previous studies on HTLV-1 have reported two types of defective proviruses [63,64]. Type 1 defective proviruses contain both 5′- and 3′-LTRs but lack a part of the proviral sequence between them, while type 2 defective proviruses lack the 5′-LTR. The defective provirus integrated in BLSC-KU1 contains both LTR regions but lacks the part between the *pol* and *env* genes, showing a similar structure to the defective HTLV-type 1 provirus. This deletion might lead to absence of BLV production in BLSC-KU1 cell lines, as previously discussed [12]; however, this cell line induced formation of leukemia in nude mice, indicating that the deletion might have significant biological functions in BLSC-KU1, such as effective escape from the host immune response. In contrast, we found the full genome of BLV provirus integrated in BLSC-KU17 cells, in agreement with a previous report of full proviral genome detection in BLSC-KU17 cell lines by Southern blotting analysis [14].

The advent of high-throughput sequencing technologies has significantly impacted biological research. High-throughput sequencing of randomly fragmented DNA has facilitated a comprehensive study of the viral structure and proviral integration sites and quantified the clonality of retrovirus-infected cells [10,22,29]. In this study, we aimed to distinguish the BLV provirus in our target samples and to identify the position of BLV insertion sites in the host genome. We thus performed PCR to amplify the BLV proviral genome and applied highly specific proviral DNA capture high-throughput sequencing to detect the proviral insertion sites in BLSC-KU1 and BLSC-KU17 cells derived from cattle with BLV-induced EBL. This information will be of importance in analyzing viral integration sites analysis in studies on BLV-induced leukemia and can also be very important for providing comprehensive information concerning BLV provirus and viral integration in these two cell lines, as the cell lines are often used in BLV-related research. The mechanisms underlying lymphoma development after BLV infection have not been fully elucidated. The proviral DNA-capture-seq method developed in the current study is very specific, highly efficient for the enrichment of target sequences, reproducible, technically easy to perform, and economically acceptable. Therefore, this method can be used to investigate the BLV integration sites in BLV-infected cattle at different stages of disease progression, such as asymptomatic carrier or persistent lymphocytes and lymphoma. Furthermore, it has the potential to be used as an innovative tool to understand the detailed mechanisms for disease progression and to screen BLV-infected cattle at risk at an earlier stage than those that have already developed lymphoma. One of the limitations of the present study was the application of this method to only two BLV-infected lymphoma cell lines; obtaining additional BLV integration site data from BLV-infected primary lymphoma samples would promote the application of the current method. Therefore, future studies to determine the BLV integration sites in a large number of BLV-infected primary lymphoma samples using this viral DNA capture library preparation method are urgently needed.

## Figures and Tables

**Table 1:** Result of viral DNA capture target enrichment high-throughput NGS sequencing system.

Sample ID	Total Reads of Viral DNA Capture NGS	Reads Mapped to FLK-BLV LTR, *Gag*, *Tax*	Ratio (%)
FLK-BLV	5,819,183	4,550,811	78.2
BLSC-KU1	5,063,009	4,083,042	80.6
BLSC-KU17	5,069,560	4,103,690	80.9

**Table 2:** Summary of the proviral structure of BLV and BLV integration sites in BLSC-KU1 and BLSC-KU17 cell lines.

Sample ID	BLV	BLV Length	BLV Strand	Integration Site (IS)	Genic/Intergenic	Total Reads
BLSC-KU1	Single copy of defective provirus	7063 bp	5′-3′ (+)	Chr19:52,448,589	Intron of RPTOR	8100
BLSC-KU17	Single copy of complete provirus	8714 bp	3′-5′ (−)	Chr9:44,084,317	Intergenic regions between RTN4IPI and ATG5	956

## Data Availability

All available data are presented in this manuscript.

## Supplementary Materials

The following are available online at: https://www.mdpi.com/article/10.3390/v14050995/s1, Table S1. List of primers used PCR amplification and sequencing of BLV provirus in KU-1 and KU-17 cell lines in this study. Table S2. Probes used in target-enrichment high-throughput NGS sequencing. Table S3. BLV integration site confirmation primers in BLSC-KU1 and BLSC-KU17 cell lines.
