# Supplementary material for: Comprehensive Comparison of Novel Bovine Leukemia Virus (BLV) Integration Sites between B-Cell Lymphoma Lines BLSC-KU1 and BLSC-KU17 Using the Viral DNA Capture High-Throughput Sequencing Method
Source: Viruses. 2022 May 7;14(5):995. doi: 10.3390/v14050995 (PMC9143949; doi:10.3390/v14050995)
Supplement: Supplementary file 1 [file viruses-14-00995-s001.zip › Supplementary Table S1. List of Primers used PCR amplification and sequencing of BLV provirus in cell lines in this study.pdf]

**Table S1.** List of Primers used PCR amplification and sequencing of BLV provirus in KU-1 and KU-17 cell lines in this study

| | | Primer ID | Binding position [1] | Sequences | Reference |
|---|---|---|---|---|---|
| PCR primers | Long PCR-1 | 94F | 94-113 | 5'-GGCTAGAATCCCCGTACCTC-3' | [31] |
| | | 8164R | 8141-8164 | 5'-GAGAGCCATTCATTTTCTAGCAGT-3' | This study |
| | Long PCR-2 | 741F | 741-765 | 5'-CCGATTTAAAGAATTACATCCATTG -3' | [31] |
| | | LTR-R | 8654-8676 | 5'-GCGAGAAACAGAAAGTAAGACAGG-3' | [31] |
| | LTR-env | 94F | 94-113 | 5'-GGCTAGAATCCCCGTACCTC-3' | [31] |
| | | 5853R | 5834-5853 | 5'-TGATCTTGCTCCAGAACGTG-3' | [31] |
| | 5'LTR | LRT-F | 2-23 | 5'-GTATGAAAGATCATGCCGACCT-3' | [31] |
| | | 807-R | 781-807 | 5'-CCAGAAGTGAAAGTCCATGGTT-3' | [31] |
| | Pol-LTR | 3802F | 3802-3825 | 5'-CCAAATACCTGTACTCTTTGCTCA-3' | This study |
| | | 8164R | 8141-8164 | 5'-GAGAGCCATTCATTTTCTAGCAGT-3' | This study |
| Sequencing Primers | | 94F | 94-113 | 5'-GGCTAGAATCCCCGTACCTC-3' | [31] |
| | | 741F | 741-765 | 5'-CCGATTTAAAGAATTACATCCATTG -3' | [31] |
| | | LTR-R | 8654-8676 | 5'-GCGAGAAACAGAAAGTAAGACAGG-3' | [31] |
| | | 5853R | 5834-5853 | 5'-TGATCTTGCTCCAGAACGTG-3' | [31] |
| | | LRT-F | 2-23 | 5'-GTATGAAAGATCATGCCGACCT-3' | [31] |
| | | 807-R | 781-807 | 5'-CCAGAAGTGAAAGTCCATGGTT-3' | [31] |
| | | pBLV-R6 | 1294-1315 | 5'-GGCCTGAAGCCAGAGGTTTTGA-3' | [32] |
| | | pBLV-R5 | 1970-1989 | 5'-GGTGTCCACAAGCATGAGGG-3' | [32] |
| | | pBLV-R4.5 | 2499-2516 | 5'-CGGAATGGGCTTTGTAAG-3' | [32] |
| | | pBLV-R3.5 | 2996-3013 | 5'-ATTGGGGATGAGATCTGC-3' | [32] |
| | | pBLV-R2.5 | 3560-3577 | 5'-GCCCTGGTGATTAAGGTC-3' | [32] |
| | | pBLV-R1.5 | 4191-4208 | 5'-ATGGGTTATATCGGCCTG-3' | [32] |
| | | pBLV-R1 | 4811-4828 | 5'-CATTTGAAGGCTTTCAGC-3' | [32] |
| | | pBLV-1st LTR-R | 1099-1115 | 5'-GGTCAGCCGGAGTAGGG-3' | [32] |

| | | | |
|---|---|---|---|
| pBLV-F0 | 4611-4631 | 5'-AGATGGGAGCTACACCATTCA-3' | [32] |
| gp51-R-5639 | 5613-5634 | 5'-AWCAACAACCTCTGGGAAGGGT-3' | [32] |
| pBLV-F1 | 5812-5830 | 5'-TCAGAGACTCACCTCCCTG-3' | [32] |
| pBLV-0R | 6055-6076 | 5'-GTCTGTAGAGACTCTTTGCGAG-3' | [32] |
| pBLV-1.5F | 6314-6331 | 5'-ATCTACTCTCACCTCTCC-3' | [32] |
| pBLV-2.5F | 6801-6818 | 5'-TTTACGCCCTGTTGCACA-3' | [32] |
| pBLV-3F | 7410-7429 | 5'-ATCAACTGGACCGCCGATGG-'3 | [32] |
| 3802F | 3802-3825 | 5'-CCAAATACCTGTACTCTTTGCTCA-3' | This study |
| pBLV-2ndLTR-F | 7919-7938 | 5'-CCAATGAACCCCCCCTTTCA-3' | This study |
| 8164R | 8141-8164 | 5'-GAGAGCCATTCATTTTCTAGCAGT-3' | This study |
| pBLV-R2 | 4027-4047 | 5'-CGAGAGTTGCAGTGGGTGAGC-3' | This study |

[1] PCR amplification and sequencing reference to FLK-BLV (EF600696)
