# Supplementary material for: Comprehensive Comparison of Novel Bovine Leukemia Virus (BLV) Integration Sites between B-Cell Lymphoma Lines BLSC-KU1 and BLSC-KU17 Using the Viral DNA Capture High-Throughput Sequencing Method
Source: Viruses. 2022 May 7;14(5):995. doi: 10.3390/v14050995 (PMC9143949; doi:10.3390/v14050995)
Supplement: Supplementary file 1 [file viruses-14-00995-s001.zip › Supplementary Table S2. Probes used for target-enrichment NGS.pdf]

Table S2. Probes used in target-enrichment high-throughput NGS sequencing [1].

**Probe ID**	**Probe Position**	**Probe sequences (5' to 3')**
Probe-LTR-1	1-100 (5'LTR), 8190-8289 (3'LTR)	TGTATGAAAGATCATGCCGACCTAGGCGCCGCCACCGCCCCGTAAACCAGACAGAGAC GTCAGCTGCCAGAAAAGCTGGTGACGGCAGCTGGTGGCTAGA
Probe-LTR-2	161-220 (5'LTR), 8350-8449 (3'LTR)	CACCTGCTGATAAATTAATAAAATGCCGGCCCTGTCGAGTTAGCGGCACCAGAAGCGT TCTTCTCCTGAGACCCTCGTGCTCAGCTCTCG GTCCTGAGCT
Probe-LTR-3	271-370 (5'LTR), 8460-8559 (3'LTR)	CGAGACCTTCTGGTCGGCTATCCGGCAGCGGTCAGGTAAGGCAAACCACGGTTTGGAG GGTGGTTCTCGGCTGAGACCACCGCGAGCTCTATCTCCGGTC
Probe-gag-1	861-960	CCACCCTAAACGAAGTGCTCTCAAACGATGGGGGCGCCCCGGGTGCATCGGCCCCAGA AGAACAACCCCCCCCTTATGACCCCCCCGCCGTTTTGCCAAT
Probe-tax-1	7941-8040	CCCCTTATCCAAACGCCCGGCCTGTCTTGGTCTGTCCCCGCGATCGACCTATTCCTAACC GGTCCCCCTTCCCCATGCGACCGGTTACACGTATGGTCCA

[1] BLV specific probes were custom-designed based on the BLV reference sequence, FLK-BLV (accession number EF600696)
