# Supplementary material for: Comprehensive Comparison of Novel Bovine Leukemia Virus (BLV) Integration Sites between B-Cell Lymphoma Lines BLSC-KU1 and BLSC-KU17 Using the Viral DNA Capture High-Throughput Sequencing Method
Source: Viruses. 2022 May 7;14(5):995. doi: 10.3390/v14050995 (PMC9143949; doi:10.3390/v14050995)
Supplement: Supplementary file 1 [file viruses-14-00995-s001.zip › Supplementary Table S3. BLV integration site confirmation primers in KU-1 and KU17 cell lines .pdf]

**Table S3.** BLV integration site confirmation primers in BLSC-KU1 and BLSC-KU17 cell lines

| Target | Primer ID | Binding position | Sequences |
|---|---|---|---|
| BLV provirus [1] | TaxendF3 | FLK-BLV nucleotide position 8618-8639 | 5'- GTCTGGCTTGCACCCGCGTTG-3' |
| | LTRendR3 | FLK-BLV nucleotide position 109-89 | 5'-TACGGGGATTCTAGCCACCAG-3' |
| KU1 genome [2] | KU1 Chr19F | Chr19:52448421-52448441 | 5'-CCTCAGATCTCCTCTACCTGG-3' |
| | KU1 Chr19R | Chr19:52448749-52448770 | 5'-GGCTGATTCGGGAGACATATAC-3' |
| KU17 genome [2] | KU17 Chr9F | Chr9:44084039-44084061 | 5'- AGCATCTTTTCTGACCACAATG-3' |
| | KU17 Chr9R | Chr9:44084339-44084361 | 5'- CCCTGAGGTTCTTCATTTCTTC-3' |
| FLK-BLV genome [3] | IS1 Chr1F | Chr1: 245436984- 245437004 | 5'-CAGCCCCAGACTCATTGTTG-3' |
| | IS2 Chr2R | Chr2:9799294-9799313 | 5'- CAAGCTCTGGGAGTGGTGAT-3' |
| | IS3 Chr9R | Chr9: 25556760- 25556779 | 5'- TTAATTCATGCGGCGCTCTG-3' |

[1] Primers targeting BLV provirus referenced FLK-BLV (accession number EF600696)

[2] BLSC-KU1 and BLSC-KU17 primers targeting host genome reference Bos_Taurus_UMD_3.1.1/Bos Tau8

[3] FLK-BLV primers targeting host genome referenced Sheep Oar_v4.0/oviAri4
